# Genetic variants of *p27 *and *p21 *as predictors for risk of second primary malignancy in patients with index squamous cell carcinoma of head and neck

**DOI:** 10.1186/1476-4598-11-17

**Published:** 2012-03-26

**Authors:** Zhongqiu Wang, Erich M Sturgis, Fenghua Zhang, Dapeng Lei, Zhensheng Liu, Li Xu, Xicheng Song, Qingyi Wei, Guojun Li

**Affiliations:** 1 Department of Head and Neck Surgery, The University of Texas MD Anderson Cancer Center, Houston, TX 77030, USA; 2 Department of Radiology, East Hospital, Tongji University School of Medicine, Shanghai 200120, China; 3 Department of Epidemiology, The University of Texas MD Anderson Cancer Center, Houston, TX 77030, USA; 4 Department of General Surgery, Hebei General Hospital, Shijiazhuang, Hebei 050051, China; 5 Department of Otolaryngology, Qilu Hospital, Shandong University, Jinan Shandong 250012, China; 6 Key Laboratory of Otolaryngology, Ministry of Health, Jinan, Shandong 250012, China; 7 Department of Otolaryngology-Head and Neck Surgery, Yuhuangding Hospital of Qingdao University, Yantai 264000, China

**Keywords:** *p21*, *p27*, Squamous cell carcinoma of head and neck, Second primary malignancy, Genetic susceptibility, Polymorphism

## Abstract

**Background:**

Cell cycle deregulation is common in human cancer, and alterations of *p27 *and *p21*, two critical cell cycle regulators, have been implicated in the development of many human malignancies. Therefore, we hypothesize that *p27 *T109G polymorphism individually or in combination with *p21 *(C98A and C70T) polymorphisms modifies risk of second primary malignancy (SPM) in patients with index squamous cell carcinoma of head and neck (SCCHN).

**Methods:**

A cohort of 1,292 patients with index SCCHN was recruited between May 1995 and January 2007 at the M.D. Anderson Cancer Center and followed for SPM occurrence. Patients were genotyped for the three polymorphisms. A log-rank test and Cox proportional hazards models were used to compare SPM-free survival and SPM risk.

**Results:**

We found that patients with *p27 *109 TG/GG, *p21 *98 CA/AA and *p21 *70 CT/TT variant genotypes had a worse SPM-free survival and an increased SPM risk than those with the corresponding *p27*109 TT, *p21 *98 CC, and *p21 *70 CC common genotypes, respectively. After combining the three polymorphisms, there was a trend for significantly increased SPM risk with increasing number of the variant genotypes (*P*_trend _= 0.0002). Moreover, patients with the variant genotypes had an approximately 2.4-fold significantly increased risk for SPM compared with those with no variant genotypes (HR, 2.4, 95% CI, 1.6-3.6).

**Conclusions:**

These results suggest that *p27 *T109G polymorphism individually or in combination with *p21 *(C98A and C70T) polymorphisms increases risk of SPM in patients with index SCCHN.

## Introduction

In the United States, squamous cell carcinoma of the head and neck (SCCHN) accounts for approximately 50,000 new cancer cases, causing 11,480 deaths annually [1,2]. SCCHN has modest survival rates mainly because of second primary malignancies (SPM), comorbid illnesses, and index cancer recurrence [3]. A leading cause of mortality in such patients is the development of SPM [3]. Additional studies on host factors that predict SPM in SCCHN patients are needed so that intensive surveillance or targeted intervention for patients at high-risk of SPM may improve prognosis. In addition to smoking, alcohol drinking and cancer treatment as risk factors for SPM, genetic predisposition might also influence the development of SPM after index SCCHN [3-10].

A common feature of human cancer is cell cycle deregulation, and cell cycle progression is governed by the activation cyclin and cyclin-dependent kinases (CDKs), which function together in the G1 phase to initiate S phase and in G2 to initiate mitosis. Through regulation of cyclin-CDK complexes, p27 and p21, two critical CDK inhibitors, act as a fail-safe mechanism involved in DNA repair and apoptosis [11]. Furthermore, p21 can inhibit CDK activity not only individually but also in combination with p27 to increase cellular levels of this inhibition [12]. Therefore, both p27 and p21 may serve as universal CDK inhibitors and play active roles in the development of SPM, because these two proteins have similar biological activities in the regulation of cell cycle control, DNA repair, and apoptosis.

Several molecular epidemiological studies have examined associations of common single nucleotide polymorphisms (SNPs) in *p21*and *p27 *with risk of various cancers, including SCCHN [13-17]. These SNPs include *p21 *C98A (a non-synonymous serine-to-arginine substitution at codon 31), *p21 *C70T (a single C-to-T substitution 20 nt downstream of the stop codon at exon 3), and *p27 *T109G (a T-to-G change at codon 109 resulting in an amino acid substitution of valine for glycine). The two polymorphisms of *p21 *have been previously reported to significantly modify risk of SPM after index SCCHN [18], but no such studies have been done for the *p27 *polymorphism. Moreover, because both p21 and p27 are involved in the same cell cycle regulation pathway and bind to cyclin D1-CDK complexes to inhibit their catalytic activity and induce cell-cycle arrest, they may have redundant or synergistic effect on cell cycle inhibition. However, there is no published study that has assessed, simultaneously, above three putatively functional SNPs in SPM. Thus, given the crucial roles of both p27 and p21 in cell cycle regulation, we hypothesize that *p27 *(T109G) polymorphism individually or in combination with *p21 *(C98A and C70T) polymorphisms modifies risk for SPM after index SCCHN, and we tested this hypothesis in a cohort of 1,292 SCCHN patients.

## Results

This was a relatively large and well-characterized cohort of 1,292 SCCHN patients who were followed up for a median follow-up time of 34 months (range 0 to 142.4 months), and the overall median follow-up time was 30.9 and 14.4 months for SPM-free patients and SPM patients, respectively. Table 1 shows demographics, tobacco smoking, alcohol drinking, and clinical variables for the patients. Of the 120 SPM patients, 81 developed SPMs at tobacco-related sites including 44 SCCHN SPM and 37 other tobacco-related cancers (34 lung cancer, 2 esophagus cancer, and 1 bladder cancer), 35 developed SPMs at non-tobacco-related SPMs (10 prostate cancer, 8 papillary thyroid carcinoma, 4 colon adenocarcinoma, 3 lymphoma, 3 hepatic adenocarcinoma, 2 breast cancer, and 1 each for the remainder including sarcoma, renal cell carcinoma, endometrial carcinoma, leukemia, and maxillary sinus adenocarcinoma); and 4 developed SPMs at both tobacco-related and non-tobacco-related sites ((2 patients with both SCCHN and prostate cancer and 2 patients with both SCCHN and papillary thyroid carcinoma).

**Table 1:** Distribution of selected characteristics of the patient cohort (n = 1,292)

	Total		SPM-Free		SPM		**P-values**^**c**^
		
Variable	**No**.	%	**No**.	%	**No**.	%	
Total patients	1,292	100	1,172	100	120	100	
Age							
≤ median (57 years)	668	51.7	629	53.7	39	32.5	< 0.001
> median (57 years)	624	48.3	543	46.3	81	67.5	
Sex							
Male	981	75.9	887	75.7	94	78.3	0.518
Female	311	24.1	285	24.3	26	21.7	
Ethnicity							
Non-Hispanic White	1,093	84.6	999	85.2	94	78.3	0.050
Other	199	15.4	173	14.8	26	21.7	
Smoking							
Never	346	26.8	321	27.4	25	20.8	0.122
Ever	946	73.2	851	72.6	95	79.2	
Alcohol							
Never	337	26.1	310	26.4	27	22.5	0.348
Ever	955	73.9	862	73.6	93	77.5	
Index Cancer Site							
Oral cavity	420	32.5	382	32.6	38	31.7	0.320
Oropharynx	575	44.5	527	45.0	48	40.0	
Larynx/Hypopharynx	297	23.0	263	22.4	34	28.3	
Index Cancer Stage							
I or II	327	25.3	295	25.0	32	26.7	0.720
III or IV	965	74.7	877	75.0	88	73.3	
Treatment							
Surgery only	230	17.8	209	17.8	21	17.5	0.894
Surgery + Adjuvant Tx^a^	322	24.9	289	24.7	33	27.5	
XRT^b^	330	25.6	302	25.8	28	23.3	
XRT + Chemotherapy	410	31.7	372	31.7	38	31.7	

^a^Adjuvant Treatment: adjuvant radiotherapy and/or chemotherapy

^b^XRT: radiotherapy

^c^*P *values were calculated from chi-square test

In this cohort of SCCHN patients, the mean age at diagnosis for the index SCCHN was 57.4 years (range, 18-94 years, median, 57 years), and the mean age of index SCCHN patients who developed SPM was significantly older than that of patients who were SPM-free (60.8 years versus 57.1 years, respectively; *P *< 0.01). The male patients (76.0%) were the majority of study subjects in this study (76.0%), but we did not find that sex was associated with SPM development (*P *= 0.518). In addition, no significant differences in characteristics regarding smoking (*P *= 0.122), alcohol drinking (*P *= 0.348), cancer site (*P *= 0.320), cancer stage (*P *= 0.720), or treatment (*P *= 0.894) were found between patients who developed SPM and those who were SPM-free. However, ethnicity was significantly different between patients who were SPM-free and patients who developed SPM, and the patients who were non-Hispanic whites more likely developed a SPM than the patients who were not non-Hispanic whites (*P *= 0.050).

The distributions of *p27 *T109G, *p21 *C98A, and *p21 *C70T genotypes between patients who developed SPM and those who did not and their associations with risk of SPM are presented in Table 2. *p27 *109 TG + GG variant genotypes were common in patients who developed SPM (62.5%) as opposed to patients who were SPM-free (43.6%), and these *p27 *109 G variant genotypes were associated with a 2-fold significantly increased risk of SPM compared with the *p27 *109 TT common homozygous genotype (HR, 2.0; 95% CI, 1.5-3.1). As we have previously reported, the variant genotypes of the two *p21 *polymorphisms were significantly associated with increased risk of SPM compared to their corresponding common homozygous genotypes after multivariable adjustment for age, sex, ethnicity, smoking, and drinking [18]. When we performed similar analyses stratified by SPM type, the effect modification of the three polymorphisms was not pronounced for tobacco-related SPMs, compared to non-tobacco-related SPMs (data not shown). Furthermore, no significant difference in SPM risk was observed, when SPMs were stratified by smoking status at the time of diagnosis of index SCCHN (ever *versus *never smokers) (data not shown). We also did not observe pronounced effects of each polymorphism of *p21 *and *p27 *on risk of SPMs at either tobacco-associated sites or non-tobacco-associated sites (data not shown).

**Table 2:** SPM risk associated with *p27/p21 *polymorphisms after index SCCHN

Genotypes	Total (No. = 1,292)		SPM-free (No. = 1,172)		SPM (No. = 120)		*P^a^*	**HR (95% CI)**^**b**^
	
	**No**.	%	**No**.	%	**No**.	%		
*P27 *T109G								
TT (Ref.^c^)	706	54.6	661	56.4	45	37.5		
TG + GG	586	45.4	511	43.6	75	62.5	< 0.001	2.0 (1.5-3.1)
*P21 *C98A								
CC (Ref.^c^)	1,105	85.5	1012	84.6	93	77.5		
CA + AA	187	14.5	160	13.6	27	22.5	0.009	1.7 (1.1-2.6)
*P21 *C70T								
CC (Ref.^c^)	706	54.6	661	56.4	45	37.5		
CT + TT	586	45.4	511	43.6	75	62.5	0.004	1.7 (1.1-2.7)

^a^χ^2 ^test for differences in the distribution of *p21/p27 *genotypes between the patients who developed SPM and the patients who did not

^b^Adjusted for age, sex, ethnicity, tobacco smoking and alcohol drinking in a Cox model

^c^Ref. = reference group

**Figure 1:**
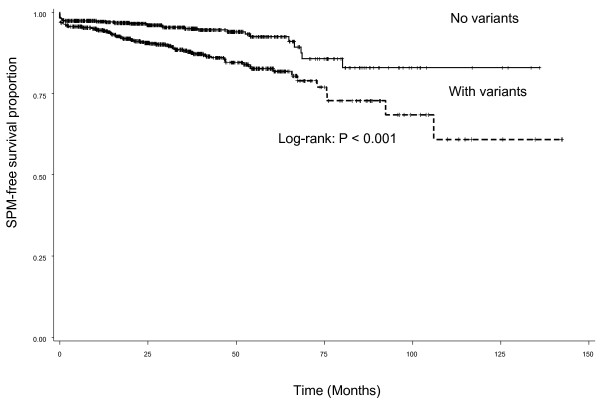
**Kaplan-Meier SPM-free survival curve stratified by combined *p21/p27 *genotypes**.

Since each of three SNPs in *p27 *and *p21 *genes in cell cycle regulation had a moderate effect on risk of SPM, we then assessed the combined effect of all three SNPs on risk of SPM in patients with index SCCHN as shown in Table 3. In order to evaluate the joint effect of these *p27 *and *p21 *polymorphisms on risk of SPM, we categorized all patients into four groups based on the number of variant genotypes they carried. We also dichotomized patients into two groups: 1) a 'no variant' reference group and 2) a 'variant' group. We found that patients who developed SPM were more likely to carry the combined variant genotypes than those who remained SPM-free (*P *< 0.001). Patients with variant genotypes of three polymorphisms in *p27 *and *p21 *experienced a significantly reduced SPM-free survival compared with those who had no variant genotypes (log-rank, *P *< 0.001, Figure 1). We also found that the SPM risk increased with the number of variant genotypes, and the trend in risk was statistically significant in a dose-response manner (*P *= 0.0002 for trend). Specifically, patients carrying 3 variant genotypes were approximately 3 times more likely to develop a SPM than those carrying no variant genotypes (HR, 3.0; 95% CI, 1.6-5.7). Those patients who possessed either variant allele (*p27 *109 G, *p21 *98 A or *p21 *70 T) were almost a 2.5 times more likely to develop a SPM (HR, 2.4, 95% CI, 1.6-3.6) than those who had the combined *p27 *109 TT, *p21 *98 CC, and *p21 *70 CC common genotypes.

**Table 3:** SPM risk associated with *p27/p21 *polymorphisms after index SCCHN

No. variant genotypes	Total (No. = 1,292)		SPM-free (No. = 1,172)		SPM (No. = 120)		*P^a^*	**HR (95% CI)**^**b**^
	
	**No**.	%	**No**.	%	**No**.	%		
0 (Ref.^c^)	606	46.9	572	48.8	34	28.3	< 0.001	1.0 (Ref.)
1	499	38.6	442	37.7	57	47.5		2.2 (1.4-3.4)
2	107	8.3	92	7.9	15	12.5		2.8 (1.5-5.3)
3	80	6.2	66	5.6	14	11.7		3.0 (1.6-5.7)
Trend								*P *= 0.0002
No variants (Ref.^c^)	606	46.9	572	48.8	34	28.3	< 0.001	1.0 (Ref.)
With variants	686	53.1	600	51.2	86	71.7		2.4 (1.6-3.6)

^a^χ^2 ^test for differences in the distribution of combined variant genotypes between the patients who developed SPM and the patients who did not

^b^Adjusted for age, sex, ethnicity, tobacco smoking and alcohol drinking in a Cox model

^c^Ref. = reference group

## Discussion and conclusion

p21 and p27 are both G1-checkpoint CDK inhibitors and have an approximately 42% amino acid homology at the amino-terminal domain, which mediates inhibition of CDK and interacts with various CDK complexes [19]. Alterations of expression or function of these two critical regulators in the cell cycle control has been implicated in the etiologies and disease prognosis of human malignancies [20]. In this study, we found that each of three SNPs of *p27 *and *p21 *genes was associated with a significantly increased risk of SPM in patients with index SCCHN. We also observed significant associations of the combined variant genotypes of three polymorphisms with increased risk of SPM in patients with index SCCHN, and the trend in risk was statistically significant in a dose-response manner. In addition, the patients with either variant allele of three SNPs were more likely to develop a SPM than the patients with no variant genotypes. Approximately 15% of SCCHN patients develop a SPM, a significant cause of posttreatment morbidity and mortality for the diseases. Although the diagnostic and therapeutic approaches, such as surgery, radiotherapy, and chemotherapy, for SCCHN patients have been improved, the poor prognosis for SCCHN patients has not significantly improved, partly because of the high frequency of SPM. Therefore, *p27 *and *p21 *polymorphisms may serve as a marker for genetic susceptibility to SPMs after index SCCHN, and for identifying high-risk subgroups of SCCHN patients who might benefit from management of alternative treatment and predictable patient outcome for an improved survival and a better quality of life. Moreover, identifying markers of risk for SPM among cancer survivors would greatly enhance secondary prevention, which is currently limited to rather simplistic clinical post-treatment screenings.

The roles of both p27 and p21 proteins in modulating cell cycle regulation has been well established. For example, overexpression of p27 was reported to inhibit CDK activation and entry into the S phase of the cell cycle [21]. As p27 may be involved in pathways regulated by both mitogenic and antiproliferative extrinsic signals, the expression level of p27 was found to be positively correlated with cell differentiation, and loss of its function may subsequently contribute to tumorigenesis [22,23]. *p27 *is rarely mutated in human malignancies; however, reduced expression of *p27 *was found to be frequent in various types of cancers, such as gastric, breast, prostate, and non-small cell lung cancers [24,25]. Furthermore, the reduced expression of p27 was also found to be correlated with poor clinical prognosis in head and neck cancers [26,27]. On the other hand, p21 protein is a downstream target of p53. In response to DNA damage, increased expression of p21 following p53 activation leads to either cell-cycle arrest at the G1 checkpoint or apoptosis. Through inhibition of proliferating cell nuclear antigen-dependent DNA replication and mismatch repair *in vitro*, p21 expression can suppress tumor growth [28]. Additionally, the overexpression of *p21 *may prevent mammalian cell proliferation and inhibit all cyclin-CDK complexes, suggesting that *p21 *is a universal inhibitor of cyclin-CDK complexes [29]. Moreover, overexpression of p21 and the subsequent overall reduced CDK activity was found to be associated with cell differentiation [30]. While somatic mutations in *p21 *gene are rare in human malignancies [31], reduced tumor expression of p21 has been associated with poor clinical prognosis [32]. Because both p27 and p21 play important roles in cell cycle control regulation, it is suggested that alterations of *p27 *and *p21 *genes, such as above mentioned genetic polymorphisms, may, at least in part, reflect increased susceptibility of SCCHN patients to SPM. Recent studies have indicated that SNPs of genes in cell cycle control play an important role in carcinogenesis and may lead to altered susceptibility to different cancers, including primary SCCHN and their SPM [13,15,17,18,33-35]. Therefore, individuals carrying polymorphic CDK inhibitors that may affect its protein function are likely to be more susceptible to cancer.

Among *p27 *and *p21 *polymorphisms, the *p27 *T109G polymorphism is within a region (amino acids 97-151) physically interacting with the Jun activation domain-binding protein 1, which triggers proteolytic degradation of p27 [36]. Therefore, it is speculated that this polymorphism may affect p27 degradation. So far, the *p27 *T109G polymorphism has been reported to be associated with risk and prognosis of prostate carcinoma and breast cancer [14-16], whereas the results were not consistent. These inconsistent results may be due to differences between studies of cancer types, patient population, and different risk factors for various types of cancers. Other factors in the studies such as small sample size, inclusion of different ethnic groups in a single study, gene-gene or gene-environment interactions, or inadequate adjustment for confounding factors could also cause the inconsistent results. The functional relevance of this *p27 *polymorphism needs further investigations as p27KIP1 exerts anti- and pro-tumorigenic activities [37]. Likewise, both *p21 *C98A and C70T polymorphisms are thought to cause p21 functional change, because *p21 *C98A (at codon 31 in exon 2) may affect the DNA-binding zinc finger motif [38], while *p21 *C70T (within the 3' untranslated region in exon 3) lies in a crucial region for cell differentiation, proliferation and tumor suppression [39,40]. Moreover, several previous studies have reported that these *p21 *polymorphisms may affect protein expression and activity and may have an effect on carcinogenesis [13,18,31]. However, the exact mechanisms for these observations remain unknown, and therefore, future studies on biological functions of these polymorphisms are needed.

Although the current study had a relatively larger cohort of SCCHN patients, there were few inherent limitations as previously described [9,10,18]. Briefly, our data on demographics, exposure, and clinical variables for the cohort were collected prospectively, but SPM outcome was recorded retrospectively. Because this cohort included multiple ethnicities, our results might not be generalizable to other ethnic populations. Additionally, because the time for SPM follow-up was limited due to the majority of patients with stage III and IV index cancer, these patients may have less opportunity to develop a SPM, and patients with late stage disease may be lost to follow-up or die relatively soon after recruitment or diagnosis. Furthermore, a screening bias for detecting tobacco-associated SPM or non-tobacco associated SPM might also exist. Finally, lack of tumor human papillomavirus (HPV) data and relatively low SPM rate might bias our estimates of association. Presently, we have not collected enough HPV data in our analysis for adjustment, and the low SPM rate might be due to a high proportion of never-smoker patients and use of strict criteria for SPM detection in this study. All of these confounding factors will be considered in our future studies.

In conclusion, our data suggest that *p27 *and *p21 *polymorphisms appear to alter individual susceptibility to SPM in patients with index SCCHN and that the *p27 *(T109G) polymorphism may individually or in combination with *p21 *(C98A and C70T) polymorphisms to increase risk of SPM in patients with an index SCCHN. However, future larger and well designed studies with longer follow-up time are needed to verify our findings.

## Materials and methods

### Study patients

In this study, patients with index SCCHN were recruited through the Head and Neck Center at the University of Texas M. D. Anderson Cancer Center between May 1995 and January 2007. Patients with SCCHN were typically followed and monitored through their treatment and post-treatment courses with regularly scheduled clinical and radiographic examinations. Details for recruitment of study patients have been previously described [9]. A SPM was carefully defined according to the modified criteria of Warren and Gates [41]. Briefly, SPMs were considered, if the second lesions had different histopathologic types, or if they developed over 5 years after treatment for the index tumor, and/or clearly separated by normal epithelium according to clinical and radiographic assessment. Pulmonary lesions were included as a SPM, if they had a non-squamous histology or if they were isolated squamous lesions over 5 years from index SCCHN and considered by both thoracic oncologist and thoracic surgeonas a SPM. If there was discrepancy or difference in opinions regarding recurrence or SPM, the second lesion was not considered a SPM but local recurrence. This study protocol was approved by the institutional review board of the University of Texas M. D. Anderson Cancer Center.

### p27 and p21 genotyping

We used genomic DNA extracted from the buffy-coat fraction of the blood samples to genotype *p27 *and *p21 *polymorphisms as previously described [13,17]. We performed the PCR analysis with a PTC-200 DNA Engine Peltier thermal cycler (MJ Research, Waltham, MA) in 10 μl of PCR mixture. The PCR mixture contained approximately 20 ng of genomic DNA, 0.1 mM dNTPs, 1× PCR buffer (50 mM KCl, 10 mM Tris HCl, and 0.1% Triton X-100), 1.5 mM MgCl_2_, 0.5 units of Taq polymerase (Sigma-Aldrich, St. Louis, MO), and 2 pmol of each primer. Specifically, for *p27 *V109G genotype, the amplification conditions were 5 min of initial denaturation at 95°C followed by 35 cycles of 15 sec at 94°C, 30 sec at 60°C, and 1 min at 72°C and a final 5-min step at 72°C for final extension. A 454-base pair (bp) PCR products were digested with restriction enzyme Bgl I at 37°C overnight (New England Biolabs, Beverly, MA), and separated with 3% agarose gel containing ethidium bromide. The genotype of codon 109 was determined by a Gly allele with a fragment length of 76, 116, and 262 bp and a Val allele with fragments lengths of 76 and 378 bp. For *p21 *C98A and *p21*C70T genotypes, the amplification conditions were 5 min of initial denaturation at 95°C; 35 cycles of 30 s at 95°C, 35 s at 55°C, and 45 s at 72°C; and a final 5-min extension step at 72°C. The conditions were the same for both genotypes except that 30 cycles of 30 s at 59°C (annealing) and 30 sec at 72°C (extension) were used for *p21*C70T. PCR products [496 base pairs (bp) for *p21 *C98A and 298 bp for *p21*C70T] were digested with the restriction enzymes *Bsm*AI (for *p21 *C98A) or *Pst*I (for *p21*C70T) (New England Biolabs, Beverly, MA) overnight at 55°C (for *Bsm*AI) or 37°C (for *Pst*I) and separated with 3% Metaphor gel containing ethidium bromide. The genotype of *p21 *C98A was identified by an A allele with fragment lengths of 77, 92, and 238 bp and a C allele with fragment lengths of 92, 165, and 238 bp. The genotype of *p21*C70T was identified by a T allele with a fragment length of 298 bp and a C allele with fragment lengths of 173 and 125 bp.

We evaluated the genotyping results without knowing the subjects with SPM or without SPM. For quality control purpose, we repeated at least 10% of random samples with a 100% concordance.

### Statistical analysis

Statistical significance was determined by *P *values (*P *< 0.05) and all tests were two-sided. The Chi-squared tests were used to exam differences in distribution of demographic, clinical, and genotyping variables between patients who had SPM and those who did not. Kaplan-Meier methods were used to determine SPM-free survival between different risk groups. Both univariate and multivariable Cox proportional hazards regression models were used for SPM assessment, and details in building the multivariable Cox models were described previously [9]. The final Cox models were fully adjusted for age, sex, ethnicity, and smoking and alcohol status after a stepwise search strategy in developing the multivariable Cox models. Software utilized for analysis was Statistical Analysis System software (SAS version 9.1.3; SAS Institute).

## Abbreviations

SCCHN: Squamous cell carcinoma of the head and neck; SPM: Second primary malignancies; HR: Hazard ratio; CI: Confidence interval; HPV: Human papillomavirus; CDKs: Cyclin-dependent kinases; SNPs: Single nucleotide polymorphisms.

## Competing interests

The authors declare that they have no competing interests.

## Authors' contributions

ZW participated in the design of the study, data analysis, and manuscript writing. EMS participated in study design, statistical analysis and manuscript writing. FZ performed literature review and drafted the manuscript. DL participated in study design and helped to draft the manuscript. ZL carried out the laboratory analysis. LX participated in statistical analysis and helped to draft the manuscript. QW participated in study design, data analysis, and manuscript writing. GL participated in study design, data analysis and manuscript writing. All authors read and approved the final manuscript.
